# Urate-lowering effects of losartan: a meta-analysis of randomised controlled trials and target trial emulation

**DOI:** 10.1038/s41440-026-02719-0

**Published:** 2026-07-15

**Authors:** Xiaoguang Xu, Ahlam Naeem, Amber Emmett, Mateusz Siedlinski, Hector Chinoy, Tomasz J. Guzik, Evangelos Kontopantelis, Maciej Tomaszewski

**Affiliations:** 1 Division of Cardiovascular Sciences, Faculty of Biology, Medicine and Health, University of Manchester, Manchester, UK; 2 Department of Internal Medicine, Jagiellonian University Medical College, Kraków, Poland; 3 Department of Rheumatology, Salford Royal Hospital, Northern Care Alliance NHS Foundation Trust, Manchester Academic Health Science Centre, Salford, UK; 4 Division of Musculoskeletal & Dermatological Sciences, Faculty of Biology, Medicine and Health, University of Manchester, Manchester, UK; 5 Centre for Cardiovascular Sciences, Queen’s Medical Research Institute, University of Edinburgh, Edinburgh, UK; 6 Center for Medical Genomics OMICRON, Jagiellonian University Medical College, Kraków, Poland; 7 Division of Informatics, Imaging & Data Sciences, Faculty of Biology, Medicine and Health, University of Manchester, Manchester, UK; 8 British Heart Foundation Manchester Centre of Research Excellence, Manchester, UK; 9 Manchester University NHS Foundation Trust Manchester, Manchester Royal Infirmary, Manchester, UK; 10 Signature Research Programme in Health Services & Systems Research, Duke-National University of Singapore, Singapore, Singapore

**Keywords:** Hyperuricaemia, Losartan, Digital hypertension, Serum urate, Uric acid, Hypertension, Target trial emulation

## Abstract

Common in hypertensive patients, hyperuricaemia is often exacerbated by guideline-recommended antihypertensive therapies such as thiazide diuretics. Losartan is known as an angiotensin II receptor type 1 antagonist with a uricosuric effect, but the magnitude of its efficacy in lowering urate has not been quantified versus a placebo-reference. We sought to quantify the urate-lowering effect of losartan versus placebo using two complementary approaches. We first conducted a meta-analysis of randomised controlled trials (RCTs) to quantify the effect of losartan on serum urate levels versus placebo. We then applied a target trial emulation framework in the UK Biobank as a secondary source of data and a replication experiment. The meta-analysis of six prospective randomised controlled trials (RCTs), including 1119 losartan-treated patients and 1093 controls, demonstrated that losartan reduced serum urate levels by approximately 0.29 mg/dL relative to placebo (95% CI: −0.46 to −0.12, *p* = 0.0009). In the target trial emulation, 23 losartan-treated individuals showed a reduction in serum urate of approximately 0.35 mg/dL (95% CI: −0.66 to −0.03, *p* = 0.03) when compared with 92 matched controls, and after accounting for 12 potential confounders including established urate-lowering therapies. Our results provide evidence for a modest yet consistent urate-lowering potential of losartan. This modest biochemical effect (~0.3 mg/dL) may be an attractive option to mitigate the diuretic-induced urate elevation. Thus, losartan can be considered as a favourable antihypertensive choice for patients managed with diuretics or those who are at risk of hyperuricaemia/gout.

**Figure:**
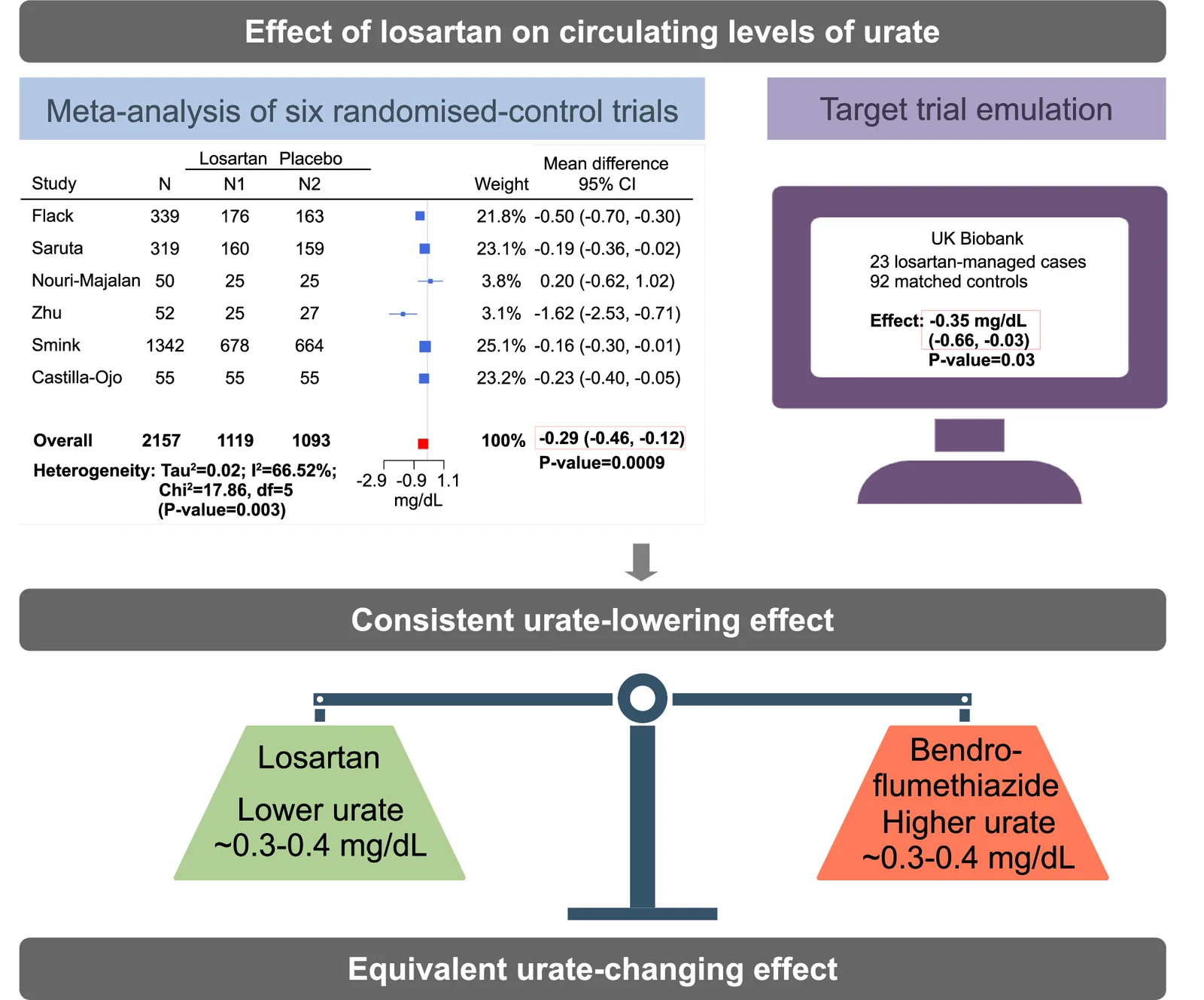

## Introduction

Approximately 25% of patients with hypertension have hyperuricaemia [1] (defined as serum urate levels greater than 7 mg/dL [2]). One of the most common causes of rise in serum urate levels in hypertensive patients is the use of thiazide diuretics [3–5]. Thiazides drive an increase in urate level by reducing its secretion and/or increasing its reabsorption in the proximal tubule of the kidneys via several transporters [i.e. organic anion transporter 1 (OAT1), organic anion transporter 4 (OAT4), multidrug resistance protein 4 (MRP4) and urate transporter 1 (URAT1) [3, 4, 6–10]] (Supplementary Fig. 1). Given that both hypertension and thiazide diuretics are recognised as independent risk factors for gout [4], both patients and prescribing clinicians are frequently reluctant to accept thiazides as an appropriate blood pressure lowering option in hypertension coexistent with hyperuricaemia/gout [11]. One of the attractive therapeutic strategies with a potential to reduce (at least to some extent) urate levels in this group of patients is selecting antihypertensives known for their urate-lowering effects, e.g. losartan. This angiotensin II type 1 receptor antagonist inhibits [12] URAT1 in the proximal tubule preventing the reabsorption of urate from the lumen of the proximal tubule cells [13, 14] (Supplementary Fig. 1). This effect on urate is a unique characteristic of losartan—other angiotensin II receptor type 1 antagonists do not influence urate metabolism [1, 2, 11, 15–19]. Due to this weak uricosuric effect, losartan is suggested as one of the therapeutic options in the British Society for Rheumatology guideline for gout [20]. Unfortunately, the objective quantitative estimates (e.g. from placebo-controlled RCTs) for urate-lowering effects of losartan are missing making it difficult for clinicians to fully utilise it for management of patients with or at risk of hyperuricaemia/gout.

In this study, we sought to quantify the urate-lowering effect of losartan using two complementary approaches. First, we conducted a meta-analysis of prospective placebo-controlled randomised controlled trials to provide the primary quantitative estimate of effect of losartan on serum urate. Second, we applied a target trial emulation framework using UK Biobank data, incorporating blood biochemistry, primary care prescription records, and clinical events to provide a complementary “real-world” evidence. By combining available biochemical measurements with prescribed medication data, along with matching and appropriate statistical adjustment, we estimated the effect of losartan on serum urate in a manner designed to emulate the comparison that would be made in a placebo-controlled trial. Collectively, our data indicate that losartan shows a modest lowering effect on serum level of urate. The magnitude of this effect is approximately equivalent (in the opposing direction) to standard antihypertensive dose of bendroflumethiazide. These data suggest, for example, that losartan may be useful in the partial neutralisation of the urate elevating effects of low-dose thiazides making it an attractive therapeutic choice in patients with or at risk of hyperuricaemia and/or gout.

Point of view

- Clinical relevance
  Losartan exerts a modest, consistent urate-lowering effect and may be a favourable antihypertensive option for patients with or at risk of hyperuricaemia or gout.
- Future direction
  Larger trials and real-world studies should determine whether losartan can effectively mitigate diuretic-induced serum urate elevations and lower the incidence of hyperuricaemia/gout in patients with hypertension.
- Consideration for the Asian population
  Evidence from Asian populations is limited—further validation in cohorts of Asian ancestry is warranted.

## Methods

### Systematic review and meta-analysis of randomised placebo-controlled clinical trials

#### Search strategy and selection criteria

The systematic review and meta-analysis were conducted using the Preferred Reporting Items for Systematic Reviews and Meta-Analyses (PRISMA) guidelines [21]. PubMed, Embase and Cochrane databases were systematically searched for relevant studies from database inception to 9th May 2024, when the last search was conducted. In addition, the reference lists of all eligible trials were manually screened to identify any relevant studies that may have been missed by the search. The electronic search strategy was developed using medical subject headings and title and abstract terms: losartan, uric acid, urate, gout, and placebo. The filters for randomised controlled trials and human studies were used as recommended by the guidelines in Chapter 4.4 of the Cochrane Handbook for Systematic Reviews of Interventions [22] to find randomised controlled trials in humans and exclude any systematic reviews and animal studies. The full electronic search strategies for all three databases were presented in the appendix (Supplementary Tables 1–3).

The PICO (population, intervention, comparison, and outcome) approach was used to guide the inclusion and exclusion criteria and the search strategy [21]. Studies were included if they assessed the effect of losartan and placebo or control group on serum urate or uric acid in adults (≥18 years old). Only published studies which described a random allocation of treatments (losartan vs placebo) to participants were eligible, including randomised cross-over trials. Furthermore, only studies measuring serum uric acid or urate levels at least one week after the initiation of losartan therapy were included [23]. There was also no limitation set on the date of publication, sample size or population demographics. Studies not in English or with only abstracts published were excluded. Animal studies were also excluded as purine metabolism varies between animals and humans [24].

### Data analysis

The search results from the PubMed, Embase and Cochrane databases were downloaded and stored in separate word documents. Next, internal duplicate studies within each database followed by external duplicates between the three databases were manually identified and removed. We identified three studies which reported on the same RENAAL trial [25–27]. The most recent post hoc analysis by Smink et al. [25] was selected to be included in the meta-analysis and the remaining duplicates were excluded.

After removal of duplicate studies, the titles and abstracts of remaining studies were initially screened against the inclusion and exclusion criteria. Studies that did not meet the inclusion criteria were excluded whilst those that met the inclusion criteria were examined in full. Studies where full articles were not available online, were requested from the University of Manchester library. Each study was examined in full and judged against the inclusion and exclusion criteria. Nine studies met the inclusion criteria. Three studies (Gansevoort et al. [28], Shahinfar et al. [29], Vogt et al. [30]) were excluded because they did not report the essential data necessary to calculate the mean change in serum uric acid level from baseline in losartan treatment arm and/or placebo group.

The key variables for data extraction included demographic information such as sample sizes, age and sex distribution of participants in total as well as in each treatment arm (losartan vs placebo/control group), the ethnicity of the participants, the length of each intervention period, the dose of losartan and the indications for treatment. Data for the predefined variables were extracted from the original publications and supplementary material of the six eligible trials [25, 31–35].

Mean differences (MDs) in change from baseline in serum uric acid between losartan and placebo were used as the effect measure for the meta-analysis. Units were consistent across trials (mg/dL), so MDs were appropriate and standardized mean differences (SMDs) were not required. Between-group MDs reported by individual trials were extracted directly; when not available, MDs were calculated from study-level group means and standard deviations.

The primary outcome was the difference in mean change in serum uric acid levels (mg/dL) from baseline between losartan treatment arm and placebo/control groups. All data on serum uric acid levels were reported in mg/dL. For studies that reported serum uric acid levels in µmol/l, the values were converted using a conversion factor of 59.48. The meta-analysis was conducted using random-effects model which used restricted maximum likelihood estimation, with inverse variance weighting, to pool the effects across studies [36]. This meta-analysis was implemented using the *Metafor* package on R (version 4.4) [37] to calculate the combined weighted MD in serum uric acid change from baseline between the placebo and losartan groups, with heterogeneity quantified by Cochran’s Q, tau^2^ and I^2^.

These data were then represented as a forest plot, generated using R (version 4.4) [37]. Publication bias was assessed using Egger’s test [38] with *p* < 0.05 indicating potential publication bias.

### Risk of bias assessment

We assessed the quality of the six eligible studies by using the revised second version of the Cochrane risk of bias tool for randomised trials (RoB2) [39]. These trials were assessed for risk of bias across 5 domains: bias due to randomisation, deviations from trial protocol, missing data and bias in measurement and reporting of outcomes. Based on this analysis, the trials were defined as either showing low risk, some concerns or high risk for each domain and for the overall risk of bias for each study. This was conducted using the criteria recommended by the Cochrane handbook [22] (Supplementary Table 4). For randomised cross-over trials, the risk of bias from carryover effects was also considered using the additional considerations suggested by the RoB2 tool [39].

### Sensitivity analysis

Since one of the studies included in the meta-analysis (Zhu et al. [34]) may be subject to a selection bias (due to its randomisation process), we conducted a sensitivity analysis excluding this study, using the same method as the primary meta-analysis. To explore potential sources of between-study heterogeneity, we conducted subgroup analyses by clinical population included (hypertension versus renal implication), losartan dose (≤50 mg versus >50 mg), and the treatment duration (≤12 weeks versus >12 weeks), as available data permitted.

### A matched cohort study in the UK Biobank

#### Target trial emulation

In this study, we adopted an analytical framework known as target trial emulation [40], which leverages the integration of temporal trait measurements with drug treatment data from large-scale biomedical resources such as the UK Biobank [41]. This strategy is designed to systematically investigate the effects of specific drug treatments on target traits, offering valuable insights into therapeutic outcomes in real-world settings. By combining clinical and temporal datasets, the target trial emulation enables robust and reproducible analyses of drug effects, effectively reducing biases and enhancing the utility of existing datasets. We followed a well-established protocol [40] to emulate the target trial of losartan treatment and serum urate levels in the UK Biobank.

### UK Biobank population

The UK Biobank is a large-scale biomedical resource that contains health-related data from over 500,000 participants, aged 37–73 years at the time of recruitment (2006–2010) [41]. This resource integrates detailed phenotypic, lifestyle, and environmental information with linked electronic health records (EHRs), genetic data, and biological samples. Longitudinal follow-up data and repeated measurements provide an opportunity for analysing temporal trends and health outcomes.

### Serum urate

Baseline and the follow-up serum urate measurements (Data Field 30880), measured by uricase PAP analysis on a Beckman Coulter AU5800, were collected from the initial assessment visit between 2006 and 2010 and the first repeat assessment visit between 2012 and 2013 (Data Field for the corresponding measurement date 53). We used the coding system lookups and mappings (Version 4) provided by UK Biobank—the code starting from “44 K” from Read Codes Version 2 or Read Codes Clinical Terms Version 3 was used to identify individuals with serum urate measurements with the corresponding measurement dates in the primary care data (Data Field 42040). The unit of measurement was converted to mg/dL using the formula: 1 mg/dL = 59.48 µmol/L.

### Losartan

Individuals treated with losartan were identified from the general practice prescription records in the primary care data (Data Field 42039). It was assumed that losartan was taken by individuals on the day of prescription.

### Eligibility criteria

**Fig. 1:**
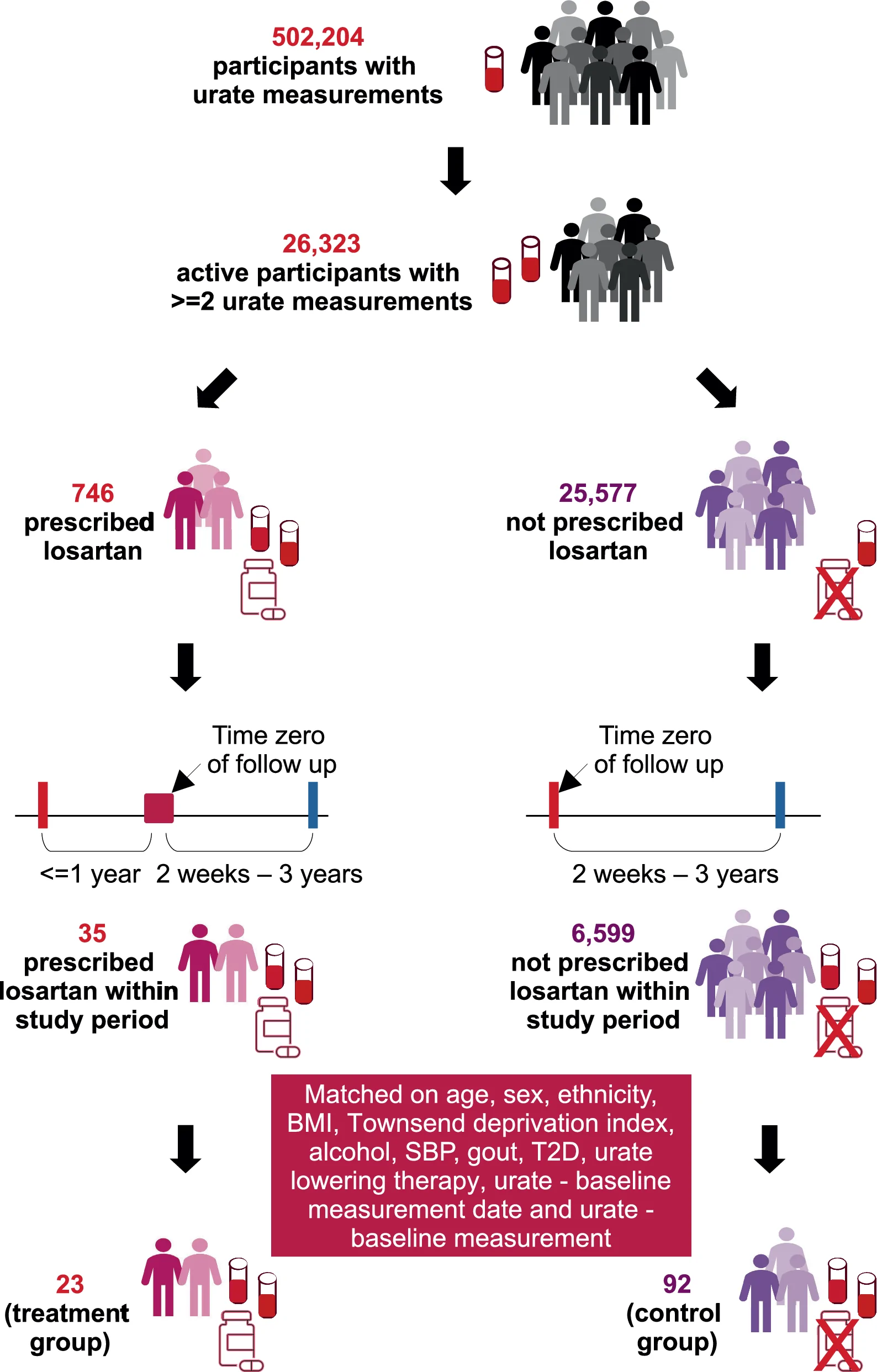
Workflow of target trial emulation sample selection in treatment and control groups. The horizontal black line represents the timeline. The vertical red and blue rectangles represent baseline and follow-up serum urate measurements respectively. The red square represents the first instance of taking losartan. BMI - body mass index, alcohol - alcohol intake frequency, SBP - systolic blood pressure at baseline, T2D - type 2 diabetes

Participants who had withdrawn their consent were excluded from the study at the general quality control level. Individuals were included if they had at least two serum urate measurements (Fig. 1). The treatment group included individuals who were new users of losartan, and the control group included individuals not prescribed losartan before and during the study period (i.e., therapy criteria) (Fig. 1). According to the experiment’s time criteria, individuals must have had at least one serum urate measurement before the treatment initiation (within one year, for the losartan group) or at the study entry (for the control group) and have at least one additional serum urate measurement taken at least two weeks after the initiation of treatment with losartan and within three years (Fig. 1). In addition, individuals with available recorded matching factors (described in assignment procedures section below) were included in this study (Fig. 1).

### Treatment strategy

The treatment strategy was defined as the effect of losartan on serum urate levels. Included in the treatment group, were individuals who started treatment with losartan during the study period. The control group consisted of individuals who had not been prescribed losartan before and during the study.

### Time zero of follow-up

Time zero of follow-up was defined as: (i) the initiation date of the first treatment with losartan (for individuals in the treatment group), and (ii) the date of the baseline serum urate level (for those in the control group).

### Follow-up period

The follow-up period was defined as the duration between time zero and the subsequent serum urate level measurement conducted within three years. For individuals with multiple follow-up measurements of serum urate, we selected the earliest one > two weeks after time zero.

### Outcome

We defined the study outcome as the change in serum urate levels (i.e., follow-up urate level minus the baseline urate level), where the baseline urate level was defined as the closest urate measurement taken within one year prior to starting losartan (for the treatment group) or the first urate measurement taken at time zero (for the control group). The follow-up urate level was defined as the closest urate level taken after a minimum of two weeks from time zero within the follow-up period.

### Assignment procedures

We applied a one-to-four genetic matching approach [42] to derive a matched control cohort of individuals not on losartan with urate-related profiles similar to the individuals on losartan (Fig. 1). Genetic matching, which refers to the Diamond & Sekhon matching algorithm, is a generalisation of propensity score and Mahalanobis distance matching, using an iterative search algorithm to maximise covariate balance between treated and the control units [42]. Matching was conducted with respect to all the matching factors defined below. To ensure a good balance, we used a propensity score caliper width of 0.01. Matching factors were selected based on their known associations with the outcome including: (i) age at recruitment (Data Field 21022), (ii) sex (Data Field 31), (iii) ethnicity (Data Field 21000), (iv) body mass index (BMI) (Data Field 21001), (v) Townsend deprivation index (Data Field 22189), (vi) alcohol intake frequency (Data Field 1558), (vii) systolic blood pressure (SBP) (Data Field 4080), (viii) type 2 diabetes (T2D), (ix) gout, (x) urate lowering therapy (allopurinol or febuxostat), (xi) baseline urate measurement date and (xii) baseline urate level. SBP was averaged using the mean of the readings if multiple measurements were available, and was further adjusted for medication use by adding 15 mmHg to the SBP (for individuals reported to be taking blood pressure–lowering medication) [43]. T2D cases were identified through triangulation of hospital admissions, death register, self-report and primary care data (Data Field 130709). Gout cases were identified following the algorithm reported previously [6]. The controls for T2D and gout were individuals not classified as cases. Individuals on non-losartan urate-lowering medication were those who have ever taken allopurinol, febuxostat, probenecid, or sulfinpyrazone reported in the primary care data (Data Field 42039).

### Analysis plan

We evaluated the quality of balance between the treated and the control groups using standardised mean difference (SMD) across each variable included in the matching process. A good balance between treated and control groups was defined as a SMD of less than 0.25 standard deviations across each matching factor as well as the propensity score [44]. Genetic matching and calculation of matching weights were performed with the R package *MatchIt* [45]. The matched dataset including the weights was then used to fit a weighted linear regression for the outcome (change in serum urate). The model used the matching weights (via the weights argument) and included the original matching covariates as regressors to improve precision and to control for any residual imbalance. Then we estimated the treatment effect using g-computation with cluster-robust standard errors, to account for pair membership [46], implemented using R package *margineffects* [47]. An alpha level of 0.05 was used throughout.

### Sensitivity analyses

To assess the robustness of the estimate for the treatment effect to influential observations in the UK Biobank target trial emulation, we conducted a leave-one-out sensitivity analysis. The analysis was repeated iteratively after excluding each treated individual and their corresponding matched control participants, while retaining the original matching structure among the remaining participants. We summarised the range of effect estimates and corresponding *P* values across all iterations.

To address a potential residual confounding arising from potential imbalances in the level of kidney function and/or diuretic use (known to influence urate and/or losartan prescribing), we derived estimated glomerular filtration rate from serum creatinine (eGFRcrea) and quantified the diuretic use in UK Biobank and included these variables in the sensitivity regression models. These models tested whether the observed association between losartan use and serum urate levels was robust to the adjustment for these factors. In line with our previous studies [48], the eGFRcrea for UKBB participants was calculated using the CKD-EPI creatinine equation in R using the ‘CKDEpi.creat’ function from the ‘*nephro*’ package. Conversion of serum creatinine (Data Field 30700) units (µmol/L) into (mg/dL) was based on dividing it by 88.4. Any eGFRcr <15 or >200 ml/min/1.73 m^2^ were set to 15 or 200 ml/min/1.73 m^2^, respectively. Individuals on thiazides or loop diuretics (i.e., diuretic use) were identified from the general practice prescription records in the primary care data (Data Field 42039).

To assess whether sequential eligibility criteria materially altered cohort composition, we compared baseline characteristics between the initially eligible and timing-eligible subsets within each treatment group. Specifically, we compared the initial losartan-treated cohort with the timing-eligible treated subset, and the initial non-losartan control cohort with the timing-eligible control subset. Baseline variables included age, sex, BMI, alcohol intake, Townsend deprivation index, systolic blood pressure, eGFRcrea, type 2 diabetes, gout, diuretic use, urate-lowering therapy, and baseline serum urate.

We also conducted a sensitivity analysis adjusting for the follow-up duration between the baseline and the first qualifying post-baseline urate measurement.

### UK Biobank ethical compliance

UK Biobank has approval from the North West Multi-centre Research Ethics Committee (MREC) to obtain and disseminate data and samples from the participants, and these ethical regulations cover the work in this study. Written informed consent was obtained from all participants.

## Results

### Losartan treatment reduces serum uric acid levels—the systematic review and meta-analysis of randomised placebo-controlled clinical trials

**Fig. 2:**
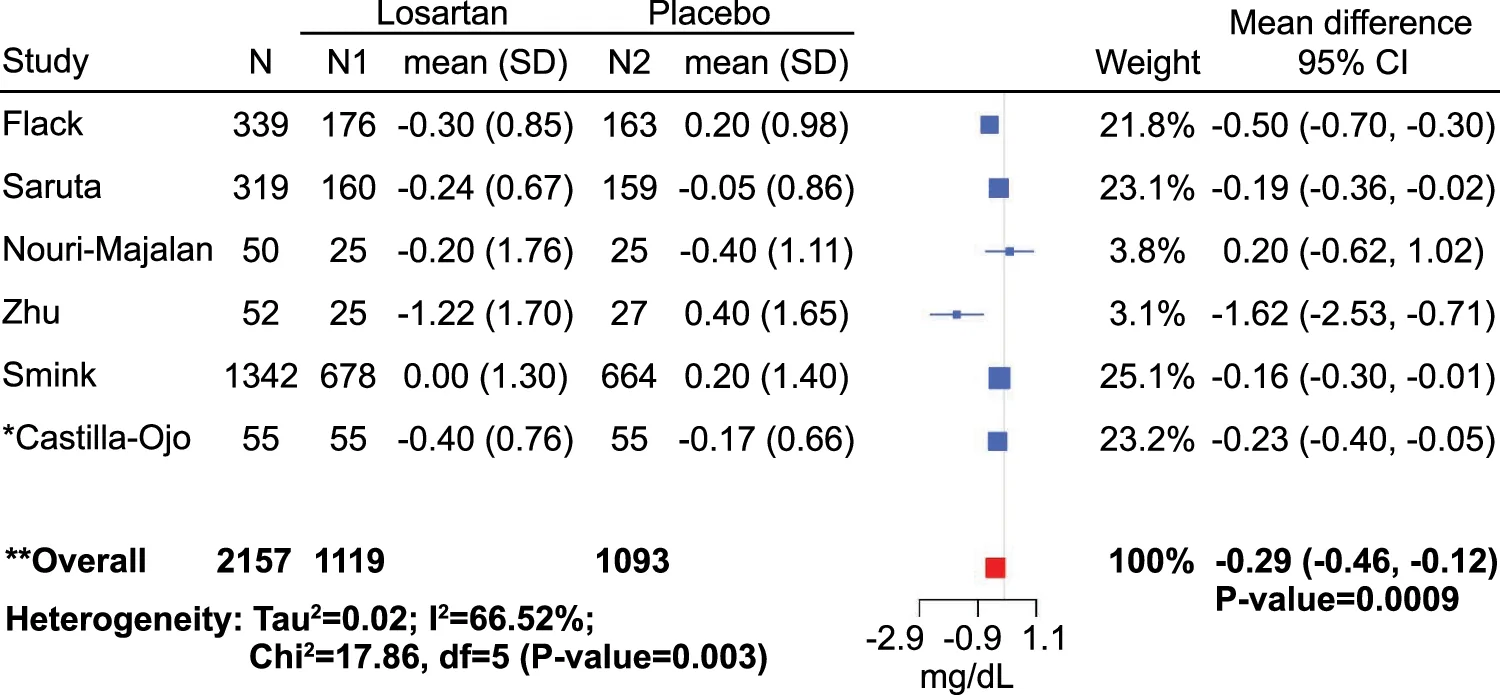
Effect of losartan on serum uric acid levels (mg/dL) compared to placebo using random-effects model. *Castilla-Ojo et al. reported serum urate levels (interchangeable with serum uric acid levels), **N represents the total number of individuals in the meta-analysis and is different from the sum of losartan-treated individuals and controls because of the cross-over nature of Castilla-Ojo et al. study. N - total sample size, N1 - sample size of the losartan group, N2 - sample size of the placebo group, SD - standard deviation, CI - confidence interval, *P* value - level of statistical significance

The process of identifying, screening and selecting studies is summarised in the PRISMA flowchart [21] (Supplementary Fig. 2, Supplementary Tables 1–3 and Supplementary Note). The total sample size was 2157, with a balanced distribution of participants between losartan treatment arm (*n* = 1119) and placebo control (*n* = 1093). The basic characteristics were comparable between both groups (Supplementary Table 5). The dose of losartan ranged from 50  to 150 mg per day and the treatment duration ranged from 4 weeks to 52 weeks. The overall risk of bias based on RoB2 tool [39] was low (Supplementary Fig. 3 and Supplementary Note). The weighted MD in serum levels of uric acid from baseline between the losartan-based treatment and placebo group was calculated at −0.29 mg/dL (95% CI: −0.46 to −0.12) (*p* = 0.0009). We used random-effects model to calculate the weighted MDs as well as the weight of each study given modest level of heterogeneity (Tau^2^ was 0.02. I^2^ was 66.52%, *p* = 0.003). Figure 2 summarises the findings of the meta-analysis while Supplementary Fig. 4 illustrates the changes from baseline in serum uric acid levels across both groups.

The subgroup analyses by clinical population, losartan dose, and the treatment duration did not identify a single source of heterogeneity, with broadly consistent direction of effect across subgroups (see Supplementary Notes).

### Losartan reduces serum urate levels—the target trial emulation in the UK Biobank

We first identified 502,204 individuals with available serum urate measurements from the UK Biobank (Fig. 1). After excluding 29 individuals who had withdrawn, 26,323 of the remaining 502,175 individuals had at least two measurements with corresponding dates (Fig. 1). Among these, 746 and 25,577 individuals were then assigned to the treatment and control groups, respectively (Fig. 1). A total of 35 individuals from the treatment group and 6,599 from the control group met further urate analysis timing criteria and were eligible for case-control matching (Fig. 1).

We then matched 23 available losartan-treated individuals to 92 control participants (with 1:4 matching ratio) based on age, sex, ethnicity, body mass index, Townsend deprivation index, alcohol intake frequency, systolic blood pressure, presence of type 2 diabetes, presence of gout, prescription of established urate-lowering medications, baseline urate measurement date and baseline urate levels (Fig. 1). The final matched cohort of 115 individuals demonstrated an adequate balance between cases and controls, with SMDs for each of the matching factors below the pre-specified threshold of 0.25 (Table 1), except for febuxostat use (SMD = 0.26). The follow-up duration was largely similar between the two groups (452.0 days in the losartan-treated group and 394.5 days in the control group, with interquartile ranges (IQRs) of 830.0 and 835.6 days, respectively).

**Table 1:** Characteristics of losartan-treated cases and matched controls

Variable	Losartan group	Control group	Standardised Mean difference
*n*	23	92	NA
Age (years)	62.09 (5.38)	62.10 (4.62)	−0.002
BMI (kg/m^2^)	29.21 (3.91)	28.54 (3.88)	0.167
Sex (male)	15 (65.2%)	60 (65.2%)	0.000
Ethnicity (white British)	22 (95.7%)	89 (96.7%)	−0.047
Ethnicity (white Irish)	1 (4.3%)	3 (3.3%)	0.065
Alcohol intake (daily or almost daily)	4 (17.4%)	13 (14.1%)	0.093
Alcohol intake (three or four times a week)	11 (47.8%)	38 (41.3%)	0.132
Alcohol intake (once or twice a week)	5 (21.7%)	27 (29.3%)	−0.019
Alcohol intake (one to three times a month)	0 (0.0%)	9 (6.6%)	−0.047
Alcohol intake (special occasions only)	1 (4.3%)	8 (8.7%)	−0.137
Alcohol intake (never)	2 (8.7%)	5 (5.4%)	0.141
Townsend deprivation index	−2.10 (3.08)	−2.37 (2.60)	0.096
Systolic blood pressure (mmHg)	154.02 (14.04)	154.78 (17.16)	−0.041
Type 2 diabetes (cases)	2 (8.7%)	13 (14.1%)	−0.171
Gout (cases)	7 (30.4%)	24 (26.1%)	0.088
Urate lowering therapy (allopurinol)	3 (13.0%)	11 (12.0%)	0.023
Urate lowering therapy (febuxostat)	1 (4.3%)	0 (0.0%)	0.261
Urate—baseline measurement date (days)	14926.57 (1076.47)	14974.03 (973.54)	−0.040
Urate—baseline measurement (mg/dL)	6.38 (1.94)	6.29 (1.69)	0.050

Data are counts and percentages or means and standard deviations

The variable urate (baseline measurement date) is expressed as the number of days since 01/01/1970

BMI - body mass index

In further sensitivity analyses of our non-losartan control cohort, the baseline characteristics were broadly similar between the initially eligible group and the timing-eligible subset, with generally small SMDs across most variables, indicating that the sequential selection process did not materially distort the cohort composition (Supplementary Table 6). In contrast, the losartan-treated cohort showed larger differences between the initially eligible group and the timing-eligible treated subset, particularly for urate-related characteristics, including gout, urate-lowering therapy, and baseline serum urate (Supplementary Table 7). These differences are clinically plausible and suggest the enrichment for participants with stronger treatment indications rather than systematic distortion of the study population.

Using linear regression analysis adjusted for all matching factors as covariates, and applying g-computation to estimate the effect of losartan on serum urate levels, we calculated a MD in serum urate levels change between losartan-treated individuals and losartan-free controls at −0.35 mg/dL (95% CI: −0.66 to −0.03; *p* = 0.03) (Supplementary Fig. 5).

In our leave-one-out sensitivity analyses, the estimated treatment effect remained consistently negative across all iterations. The effect estimates ranged from −0.28 to −0.41 mg/dL, with corresponding *P* values ranging from 0.010 to 0.080 (Supplementary Table 8). The direction of the effect did not change in any iteration, and the majority of estimates remained statistically significant.

Additional sensitivity analyses showed that the association remained statistically significant after additional adjustment for estimated glomerular filtration rate (based on serum creatinine) alone (*p* = 0.041) or the diuretic use alone (*p* = 0.049), and was attenuated, but remained consistent in the direction and magnitude, when both variables were included in the model (*p* = 0.059) (see Supplementary Table 9).

Finally, in a sensitivity analysis dedicated to the follow-up duration, the association between losartan use and the lower serum urate remained statistically significant (effect = −0.35, 95% CI −0.68 to −0.01 and *p* = 0.045). No association was observed between follow-up duration and change in serum urate (*p* = 0.08).

## Discussion

Our study provides a robust quantification of urate-lowering effect of losartan derived from two independent and complementary sources of information – a meta-analysis of placebo-controlled randomised clinical trials and a target trial emulation. Both experiments showed that losartan lowers serum levels of urate and quantified the magnitude of its placebo-controlled effect approximately equivalent (in the opposing direction) to standard antihypertensive dose of bendroflumethiazide. It is important to note that this effect represents only approximately 14–17% and 10–12% of the average urate-lowering effect of allopurinol and febuxostat, respectively [3, 49]. Consequently, losartan is not a substitute for these established urate-lowering therapies (ULT). Instead, this modest urate-lowering potential positions losartan as an antihypertensive agent: (i) with a potential for additional marginal urate reduction in hypertensive patients who have not yet reached the recommended treatment targets [20] on ULT, (ii) mitigating urate-elevating effects of diuretics. Indeed, there is a dose-dependent relationship between bendroflumethiazide and the rise in serum levels of urate (Supplementary Fig. 1, Supplementary Fig. 6)—a daily dose of 2.5 mg of this thiazide [50] is associated with an increase in urate of approximately 0.4 mg/dL compared to placebo (Supplementary Fig. 1, Supplementary Fig. 6). Our findings suggest that choosing losartan as an antihypertensive partner in these patients may partially neutralise this urate elevation. Only few studies have examined the effects of losartan on uric acid/urate when prescribed together with diuretics. Shahinfar et al. [29] found that in patients with thiazide-induced hyperuricaemia, losartan indeed reduced serum uric acid levels but this effect was sustained for only approximately three weeks. Soffer et al. [51] noted a dose-dependent decrease in serum levels of uric acid when 25 mg, 50 mg and 100 mg of losartan were given alongside 25 mg of hydrochlorothiazide. Further studies are needed to comprehensively evaluate the clinical advantage of losartan’s urate-lowering effect in patients on thiazides to establish whether losartan acts as a partial counterbalance to thiazides and is effective in reducing or delaying the appearance and/or flare ups of gout.

While urate lowering potential has been recognised by previous studies [1, 16–19] there was a lack of robust clinically relevant quantification of losartan’s effect on urate/uric acid versus placebo—previous meta-analyses have focused primarily on comparing losartan versus other medications. These estimates are much more difficult to interpret and integrate in clinical practice than those from a comparison with a placebo control. Whether the urate lowering potential of losartan depends on the dose is not entirely clear—half of the studies included in the meta-analysis [31, 32, 35] showed a reduction in serum levels of uric acid at a dose of losartan as low as 50 mg. Our results are consistent with this body of evidence and suggest that the urate-lowering effect is broadly similar across the dose range studied, with no evidence of a significant dose-response relationship. Importantly, our meta-analysis integrated trials conducted across clinically diverse settings, including essential hypertension, diabetic nephropathy, post–renal transplantation, and diet-interventions. This diversity likely contributed to the observed between-study heterogeneity and suggests that the pooled estimate should be interpreted as an average effect across heterogeneous clinical populations. Our subgroup analyses by clinical population, losartan dose, and the treatment duration did not identify a single dominant source of heterogeneity, although the smaller subgroups were imprecisely estimated. Accordingly, the findings support a broadly consistent urate-lowering effect of losartan, but generalisability to any one specific clinical context should be done cautiously.

We should also acknowledge the feasibility, benefits and limitations of the target trial emulation framework we adopted in this analysis. Traditional RCTs are often constrained by costs, operational difficulties, small sample sizes, narrowly defined participant groups and safety concerns [48, 52]. The UK Biobank’s extensive clinical information, including primary care prescription data, offers an opportunity to examine medication effects in real-world settings with a high level of control. Indeed, leveraging information from patient records and detailed medication data we have managed to match patients on losartan with suitable controls across 12 clinical dimensions known to associate with urate including established urate-lowering medications such as allopurinol and febuxostat. It is not always feasible to achieve this level of clinical similarity between treatment arms in traditional RCTs. This approach enables systematic outcome analysis by combining real-world data with the robust statistical methodologies applied by randomised controlled trials.

Several limitations of the target trial emulation should be noted. First, the number of treated individuals was relatively small (*n* = 23), which reduces statistical power and may increase the risk of bias due to residual confounding and model instability. As the risk mitigation strategies, our matching achieved acceptable balance under the conventional thresholds, and we applied several strategies to assess the robustness of our findings. Although the confidence interval of the primary estimate was relatively wide and close to the null, the direction and magnitude of the effect were consistent with those observed in the RCT meta-analysis. The leave-one-out sensitivity analysis further demonstrated that the findings were not driven by any single matched set and were reasonably robust to the influential observations.

Second, we acknowledge that a residual confounding is an important limitation of the UK Biobank analysis. Although we adjusted for 12 key covariates in the baseline model, we sought further insights from additional sensitivity analyses inclusive of eGFRcrea and diuretic use given their known associations with urate and/or losartan prescribing. The modest attenuation in the statistical significance of the estimate most likely reflects the increased uncertainty driven by additional covariates in a model based on the small sample size, rather than an elimination of the treatment effect. Although some potential confounders including other medications known to influence urate levels such as aspirin and SGLT2 inhibitors together with dietary intake, and weight fluctuations could not be fully accounted for in our analysis, we are satisfied that the key clinical variables were adjusted for in the analysis. Further studies with larger sample sizes and inclusive of detailed information on modifiers of serum urate are warranted.

Third, the target trial emulation incorporated a broad range of follow-up durations, reflecting the real-world clinical practice, and we used the earliest qualifying post-baseline urate measurement in our analysis. Reassuringly, the median follow-up time was comparable between the groups, and the adjustment for follow-up duration did not materially alter the direction or significance of the observed association. Although some residual variability related to the outcome timing may remain, these findings support the general robustness of our results across differing follow-up time intervals. Fourth, we further acknowledge that in the UK Biobank target trial emulation, the comparator to active treatment is defined not as a literal placebo group, but as a cohort of non-losartan users.

Fifth, our target trial emulation required at least two serum urate measurements within a defined time window, which may have preferentially selected individuals with hyperuricaemia and gout. Consequently, the estimated urate-lowering effect of losartan may be most generalisable to such patients and/or those undergoing routine laboratory monitoring, rather than to all treated individuals in the general population. With these limitations in mind, we conclude that while the UK Biobank target trial emulation delivers supportive, real-world evidence, we acknowledge that the results from this experiment should be interpreted as complementary and hypothesis-generating rather than definitive.

The quantitative evidence for modest urate-lowering effect of losartan has been derived primarily from cohorts of non-Asian ancestry. While individuals recruited in China and Japan contributed to the meta-analysis of RCTs, they represented only a small fraction of the informative data. Further investigations within Asian populations are required to substantiate the clinical relevance and magnitude of this effect; especially given the disproportionately higher rates of hyperuricaemia and gout in Asian relative to European populations.

Overall, the RCT meta-analysis provides the primary source of the quantitative evidence, while the UK Biobank target trial emulation offers complementary real-world level of support. The consistency of findings across these two analytically distinct approaches strengthens the evidence for modest yet reproducible urate-lowering effect of losartan. This triangulation also suggests that combining a placebo-controlled meta-analysis with a target trial emulation may be a useful and cost-effective strategy for evaluating the metabolic effects of commonly prescribed drugs.

## Supplementary information

Supplementary material
