# Supplementary material for: Urate-lowering effects of losartan: a meta-analysis of randomised controlled trials and target trial emulation
Source: Hypertens Res. 2026 Jul 15;49(9):2486–98. doi: 10.1038/s41440-026-02719-0 (PMC13547104; doi:10.1038/s41440-026-02719-0)
Supplement: Supplementary file 1 [file 41440_2026_2719_MOESM1_ESM.docx]

**Urate-lowering effects of losartan: a meta-analysis of randomised controlled trials and target trial emulation**

**Supplementary material**

Xiaoguang Xu^1^*, Ahlam Naeem^1^*, Amber Emmett^1^, Mateusz Siedlinski^2^,

Hector Chinoy^3,4^, Tomasz J Guzik^2,5,6^, Evan Kontopantelis^7,8^,

Maciej Tomaszewski^1,8,9,10^

^1^Division of Cardiovascular Sciences, Faculty of Biology, Medicine and Health, University of Manchester, Manchester, UK

^2^Department of Internal Medicine, Jagiellonian University Medical College, Kraków, Poland

^3^Department of Rheumatology, Salford Royal Hospital, Northern Care Alliance NHS Foundation Trust, Manchester Academic Health Science Centre, Salford, United Kingdom

^4^Division of Musculoskeletal & Dermatological Sciences, Faculty of Biology, Medicine and Health, University of Manchester, Manchester, UK

^5^Centre for Cardiovascular Sciences, Queen’s Medical Research Institute, University of Edinburgh, Edinburgh, UK

^6^Center for Medical Genomics OMICRON, Jagiellonian University Medical College, Kraków, Poland

^7^Division of Informatics, Imaging & Data Sciences, Faculty of Biology, Medicine and Health, University of Manchester, Manchester, UK

^8^British Heart Foundation Manchester Centre of Research Excellence, Manchester, UK

^9^Manchester University NHS Foundation Trust Manchester, Manchester Royal Infirmary, Manchester, UK

^10^Signature Research Programme in Health Services & Systems Research, Duke-National University of Singapore, Singapore

Correspondence:

Professor Maciej Tomaszewski, Division of Cardiovascular Sciences, Faculty of Biology, Medicine and Health, University of Manchester, Manchester, UK

E-mail: [maciej.tomaszewski@manchester.ac.uk](mailto:maciej.tomaszewski@manchester.ac.uk)

*These authors contributed equally.

**Table of Contents**

[**Supplementary Notes**	5](#_Toc226719758)

[Calculation of combined means and standard deviations	5](#_Toc226719759)

[Literature search, screening and randomised trials included in the meta-analysis	6](#_Toc226719760)

[Publication bias assessment	7](#_Toc226719761)

[Risk of bias assessment	7](#_Toc226719762)

[Sensitivity analysis to quantify the effect of losartan on serum uric acid through meta-analysis	8](#_Toc226719763)

[Subgroup analyses by clinical population included, losartan dose and the treatment duration	8](#_Toc226719764)

[References	9](#_Toc226719765)

[**Supplementary Figures**	11](#_Toc226719766)

[Supplementary Figure 1. Effect of losartan and thiazides on urate transporters in the proximal convoluted tubules.	11](#_Toc226719767)

[Supplementary Figure 2. PRISMA flowchart summarising the screening and selection of eligible studies.	12](#_Toc226719768)

[13](#_Toc226719769)

[Supplementary Figure 3. Risk of bias in the 6 eligible studies determined using the revised Cochrane risk of bias tool (RoB2).	13](#_Toc226719770)

[Supplementary Figure 4. Mean change in serum uric acid levels (mg/dL) and standard error in losartan (orange) and placebo (blue) groups of trials included in meta-analysis.	14](#_Toc226719771)

[Supplementary Figure 5. Drug effects on serum urate levels.	15](#_Toc226719772)

[Supplementary Figure 6: Mean change in serum uric acid levels (mg/dL) and standard error with placebo and different doses of Bendroflumethiazide (1.25, 2.5, 5 and 10 mg/day).	16](#_Toc226719773)

[**Supplementary Tables**	17](#_Toc226719774)

[Supplementary Table 1. Criteria for determining the overall risk of bias in each study based on the risk of bias in each domain.	17](#_Toc226719775)

[Supplementary Table 2. Search strategy for PubMed.	18](#_Toc226719776)

[Supplementary Table 3. Search strategy for Embase.	20](#_Toc226719777)

[Supplementary Table 4. Search strategy for Cochrane.	22](#_Toc226719778)

[Supplementary Table 5. Summary table of trials included in the meta-analysis.	23](#_Toc226719779)

[Supplementary Table 6. Characteristics of the initial control group and the timing-eligible control subset.	25](#_Toc226719780)

[Supplementary Table 7. Characteristics of the initial treatment group and the timing-eligible treated subset.	27](#_Toc226719781)

[Supplementary Table 8. Effects of losartan on serum urate from the leave-one-out analysis of the UK Biobank target trial emulation.	29](#_Toc226719782)

[Supplementary Table 9. Effects of losartan on serum urate with additional adjustment for renal function and diuretic use in the UK Biobank target trial emulation – sensitivity analyses.	31](#_Toc226719783)

# **Supplementary Notes**

## Calculation of combined means and standard deviations

In three studies, including Flack et al^1^, Saruta et al^2^, and Zhu et al^3^, not all participants completed the study. To reflect the sample size that completed the study (those whose end-study serum uric acid levels were reported), mean and sex distribution were re-calculated using the equations recommended for combining statistics from two groups in Chapter 6.5.2.10 of the Cochrane Handbook for Systematic Reviews of Interventions^4^. The following equations^4^ were used to calculate the combined mean and standard deviations for the total sample that completed the study:

Mean	$\frac{N_{1}M_{1}+N_{2}M_{2}}{N_{1}+N_{2}}$

Standard Deviation $\sqrt{\frac{{(N}_{1}-1){SD}_{1}^{2}+{(N}_{2}-1){SD}_{2}^{2}+\frac{N_{1}N_{2}}{N_{1}+N_{2}}(M_{1}^{2}+M_{2}^{2}-2M_{1}M_{2})}{N_{1}+N_{2}-1}}$

losartan group *(N_1_ = sample size who completed the study, M_1_= mean, SD_1_= standard deviation),* placebo group *(N_2_ = sample size who completed the study, M_2_ = mean, SD_2_= standard deviation).*

Where provided, changes in serum uric acid levels from baseline in placebo and losartan groups, alongside their standard deviations (SD), were extracted from the eligible studies. Whilst four studies^1,2,5,6^ reported on this directly, two studies^3,7^ only reported mean serum uric acid levels and SD at baseline and after treatment with either placebo or losartan. The difference between the baseline and post intervention serum uric acid levels was calculated to determine the mean change in serum uric acid from baseline for both placebo and losartan groups in both studies. The standard deviations for the mean change were also calculated for each group using the following equation:

$$SD= \sqrt{A^{2}+ B^{2}-2r(AB)}$$

*SD = standard deviation. A = standard deviation for baseline serum uric acid levels, B = standard deviation for post intervention serum uric acid levels, r = correlation 0.6.*

## Literature search, screening and randomised trials included in the meta-analysis

185 records were identified from PubMed (41), Embase (81) and Cochrane (63); 80 duplicates were removed and the titles/abstracts of the remaining 105 studies were screened following PRISMA 2020 Record screen guidelines^8^. Of these, 79 studies did not meet the inclusion criteria and one publication was only available as an abstract. A total of 25 studies were examined in full: (i) 13 were then excluded as they did not compare losartan to placebo; (ii) two studies^9,10^ reported the results of the same trial; (iii) one study was removed as it measured serum uric acid levels only six hours post losartan administration (iv) three studies^11–13^ were removed due to unavailability of key information and (v) one study^14^ was excluded as it was not a RCT. The reference lists of five eligible trials were then manually searched and one additional study^1^ was identified from the reference lists. After full assessment six randomised studies^1–3,5–7^ were retained for the meta-analysis (Table S4). All studies included in the meta-analysis used a randomised process to allocate patients to either losartan-based treatment or placebo/control group. Four studies^1,2,5,7^ were randomised controlled parallel group trials. One study^3^ was a randomised case-control study, and another study^6^ was a randomised cross-over trial.

## Publication bias assessment

Egger’s test did not indicate the presence of publication bias (p = 0.1654). Although formal assessment of publication bias using Egger’s test is limited by the small number of included studies (n = 6), the consistency of effects across trials provides some reassurance regarding the robustness of the pooled estimate.

## Risk of bias assessment

The RoB2 tool^15^ was used to assess the quality of the six eligible studies: (i) four studies^1,2,5,6^ had a low risk of bias; (ii) one study^7^ showed some concerns and (iii) one study^3^ was considered to have a high risk of bias. This is summarised in Figure S3, generated using Robvis tool^16^.

The trial from Zhu et al^3^ was classified to have a high risk of bias due to their randomisation process. This is because they randomised patients to the placebo or losartan group based on the parity of their identification (ID) number^3^. RoB2 tool^15^ classifies this method of randomisation as involving high risk of bias as investigators may predict the allocation sequence of participants to each intervention arm before participants are fully recruited for the study. This may cause selection bias^15^. The trial from Nouri-Majalan et al^7^ showed some concerns for selection bias. This is because they did not provide information regarding whether the allocation sequence was adequately concealed from the investigators recruiting the participants until all the participants had been enrolled^17^. Attempts to contact the authors for more information were unsuccessful.

## Sensitivity analysis to quantify the effect of losartan on serum uric acid through meta-analysis

One of the studies included in the meta-analysis may have selection bias (Zhu et al^3^) due to their randomisation process. However, this study contributes a small percentage (3.1%) to the summary statistic. Sensitivity analysis excluding this study from the meta-analysis still suggests that losartan significantly reduces serum uric acid levels compared to a placebo (p = 0.0006).

## Subgroup analyses by clinical population included, losartan dose and the treatment duration

Our subgroup analyses did not identify a single statistically significant source of heterogeneity including clinical population (p-value for subgroup difference = 0.854) or losartan dose (p-value for subgroup difference = 0.99). The subgroup analysis stratified on the treatment duration yielded the same grouping structure as the clinical phenotype analysis, suggesting that the duration of treatment with losartan is unlikely to explain the observed heterogeneity.

In the clinical population subgroup analysis, the pooled effect was −0.30 mg/dL (95% CI −0.49 to −0.12, p-value = 0.001) in hypertension studies and −0.48 mg/dL (95% CI −1.49 to 0.53, p-value = 0.350) in renal implication studies. In the subgroup analysis stratified on losartan dose, the pooled effect was −0.39 mg/dL (95% CI -0.99 to 0.22, p-value = 0.213) for doses ≤50 mg and −0.32 mg/dL (95% CI -0.66 to 0.010, p-value = 0.057) for doses >50 mg.

## References

1	Flack JM, Saunders E, Gradman A, Kraus WE, Lester FM, Pratt JH, et al. Antihypertensive efficacy and safety of losartan alone and in combination with hydrochlorothiazide in adult African Americans with mild to moderate hypertension. *Clin Ther* 2001; **23**: 1193–1208. doi:10.1016/s0149-2918(01)80101-6

2	Saruta T, Ogihara T, Matsuoka H, Suzuki H, Toki M, Hirayama Y, et al. Antihypertensive efficacy and safety of fixed-dose combination therapy with losartan plus hydrochlorothiazide in Japanese patients with essential hypertension. *Hypertens Res* 2007; **30**: 729–739. doi:10.1291/hypres.30.729

3	Zhu X, Chen J, Han F, Cheng M, Xu L, Zhang L, et al. Efficacy and safety of losartan in treatment of hyperuricemia and posttransplantation erythrocytosis: results of a prospective, open, randomized, case-control study. *Transplant Proc* 2009; **41**: 3736–3742. doi:10.1016/j.transproceed.2009.06.225

4	Cochrane Handbook for Systematic Reviews of Interventions. https://training.cochrane.org/handbook. Accessed 2 December 2024

5	Smink PA, Bakker SJL, Laverman GD, Berl T, Cooper ME, de Zeeuw D, et al. An initial reduction in serum uric acid during angiotensin receptor blocker treatment is associated with cardiovascular protection: a post-hoc analysis of the RENAAL and IDNT trials. *J Hypertens* 2012; **30**: 1022–1028. doi:10.1097/HJH.0b013e32835200f9

6	Castilla-Ojo N, Turkson-Ocran R-A, Conlin PR, Appel LJ, Miller ER, Juraschek SP. Effects of the DASH diet and losartan on serum urate among adults with hypertension: Results of a randomized trial. *J Clin Hypertens (Greenwich)* 2023; **25**: 915–922. doi:10.1111/jch.14721

7	Nouri-Majalan N, Ghafari A, Moghaddasi S. Effect of angiotensin II type-1 receptor blockers on stable allograft kidneys: prospective randomized study. *Transplant Proc* 2009; **41**: 2832–2834. doi:10.1016/j.transproceed.2009.07.021

8	Page MJ, McKenzie JE, Bossuyt PM, Boutron I, Hoffmann TC, Mulrow CD, et al. The PRISMA 2020 statement: An updated guideline for reporting systematic reviews. *PLoS Med* 2021; **18**: e1003583. doi:10.1371/journal.pmed.1003583

9	Miao Y, Ottenbros SA, Laverman GD, Brenner BM, Cooper ME, Parving H-H, et al. Effect of a reduction in uric acid on renal outcomes during losartan treatment: a post hoc analysis of the reduction of endpoints in non-insulin-dependent diabetes mellitus with the Angiotensin II Antagonist Losartan Trial. *Hypertension* 2011; **58**: 2–7. doi:10.1161/HYPERTENSIONAHA.111.171488

10	Appel GB, Radhakrishnan J, Avram MM, DeFronzo RA, Escobar-Jimenez F, Campos MM, et al. Analysis of metabolic parameters as predictors of risk in the RENAAL study. *Diabetes Care* 2003; **26**: 1402–1407. doi:10.2337/diacare.26.5.1402

11	Gansevoort RT, de Zeeuw D, Shahinfar S, Redfield A, de Jong PE. Effects of the angiotensin II antagonist losartan in hypertensive patients with renal disease. *J Hypertens Suppl* 1994; **12**: S37-42.

12	Shahinfar S, Simpson RL, Carides AD, Thiyagarajan B, Nakagawa Y, Umans JG, et al. Safety of losartan in hypertensive patients with thiazide-induced hyperuricemia. *Kidney Int* 1999; **56**: 1879–1885. doi:10.1046/j.1523-1755.1999.00739.x

13	Vogt L, Waanders F, Boomsma F, de Zeeuw D, Navis G. Effects of dietary sodium and hydrochlorothiazide on the antiproteinuric efficacy of losartan. *J Am Soc Nephrol* 2008; **19**: 999–1007. doi:10.1681/ASN.2007060693

14	Nakashima M, Uematsu T, Kosuge K, Kanamaru M. Pilot study of the uricosuric effect of DuP-753, a new angiotensin II receptor antagonist, in healthy subjects. *Eur J Clin Pharmacol* 1992; **42**: 333–335. doi:10.1007/BF00266358

15	Sterne JAC, Savović J, Page MJ, Elbers RG, Blencowe NS, Boutron I, et al. RoB 2: a revised tool for assessing risk of bias in randomised trials. *BMJ* 2019; **366**: l4898. doi:10.1136/bmj.l4898

16	McGuinness LA, Higgins JPT. Risk-of-bias VISualization (robvis): An R package and Shiny web app for visualizing risk-of-bias assessments. *Res Synth Methods* 2021; **12**: 55–61. doi:10.1002/jrsm.1411

17	Kim J, Shin W. How to do random allocation (randomization). *Clin Orthop Surg* 2014; **6**: 103–109. doi:10.4055/cios.2014.6.1.103

# **Supplementary Figures**

## Supplementary Figure 1. Effect of losartan and thiazides on urate transporters in the proximal convoluted tubules. The green solid line represents stimulation, and the red dashed line with a cross represents inhibition. OAT1 – organic anion transporter 1, OAT4 – organic anion transporter 4, NPT1 – sodium dependent phosphate transporter type 1, ABCG2 – ATP-binding cassette G member 2, MRP4 – multidrug resistance protein 4, PCT – proximal convoluted tubule, URAT1 – urate transporter 1, GLUT9 – glucose transporter 9.

**Identification of studies via databases and registers**

Reports not retrieved: Only abstract published (n=1)

Records excluded:

(n=79)

Records screened (n=105)

Records identified from: n=185

- PubMed (n=41)
- Embase (n=81)
- Cochrane (n=63)

Records removed *before screening*:

Duplicate records removed

(n=80)

**Identification**

Reports sought for retrieval (n=26)

**Screening**

Reports assessed for eligibility (n=25)

Reports excluded:

Losartan not compared to placebo (n=13)

Report on same trial (n=2)

<1week (n=1)

Data not retrieved (n=3)

Not randomised (n=1)

Eligible studies (n=5)

References (n=1)

**Included**

Total studies in review (n=6)

## Supplementary Figure 2. PRISMA flowchart summarising the screening and selection of eligible studies.

## Supplementary Figure 3. Risk of bias in the 6 eligible studies determined using the revised Cochrane risk of bias tool (RoB2). Figure generated using Robvis tool. Green (low risk of bias), yellow (some concerns), red (high risk of bias). D1 – bias from randomisation process, D2 – bias from deviation from intended interventions, D3 – bias from missing data, D4 – bias in measuring the outcome, D5 – bias in reporting outcomes.

Mean change in SUA (mg/dL)

|  | Placebo | Losartan |
| --- | --- | --- |
| Flack (2001) | 0.2 | -0.3 |
| Saruta (2007) | -0.05 | -0.24 |
| Nouri-Majalan (2009) | -0.4 | -0.2 |
| Zhu (2009) | 0.4 | -1.22 |
| Smink (2012) | 0.2 | 0 |
| Castilla-Ojo (2023) | -0.17 | -0.4 |

## Supplementary Figure 4. Mean change in serum uric acid levels (mg/dL) and standard error in losartan (orange) and placebo (blue) groups of trials included in meta-analysis.

SUA – serum uric acid, Mean change – mean serum uric acid level (follow-up) minus mean serum uric acid level (baseline).

## Supplementary Figure 5. Drug effects on serum urate levels. Effects of bendroflumethiazide are based on the study by Carlsen et al. (BMJ. 1990;300:975-978). Bfz 1.25mg – bendroflumethiazide - dose of 1.25 mg/day, Bfz 2.5mg – bendroflumethiazide - dose of 2.5 mg/day, UKB – target trial emulation using the UK Biobank cohort, meta – meta-analysis of randomised controlled trials.

Mean change in SUA (mg/dL)

|  | Urate change at 10 weeks (mg/dL) |
| --- | --- |
| Placebo | -0.08 |
| 1.25 mg/day | 0.32 |
| 2.5 mg/day | 0.49 |
| 5 mg/day | 1.06 |
| 10 mg/day | 1.14 |

## Supplementary Figure 6: Mean change in serum uric acid levels (mg/dL) and standard error with placebo and different doses of Bendroflumethiazide (1.25, 2.5, 5 and 10 mg/day). SUA – serum uric acid. Mean change – mean serum uric acid level (baseline) minus mean serum uric acid level (after 10 weeks of treatment). Data are based on the study by Carlsen et al. (BMJ. 1990;300:975-978).

# **Supplementary Tables**

## Supplementary Table 1. Criteria for determining the overall risk of bias in each study based on the risk of bias in each domain. Adapted from the Cochrane Handbook for Systematic Reviews of Interventions.

| **Combined risk of bias Criteria** | |
| --- | --- |
| Low risk | All domains classified as low risk |
|  |  |
| Some concerns | At least one domain classified as showing some concerns |
|  |  |
| High risk | More than one domain classified as showing some concerns  OR  At least one domain classified as high risk |

## Supplementary Table 2. Search strategy for PubMed. [mh] = medical subject heading, [tiab] = title/abstract, [pt] = publication type, [ti] = title.

| **PubMed** | |
| --- | --- |
| 1 | Losartan [mh] or Losartan [tiab] |
| 2 | Uric acid [mh] or uric acid [tiab] or urate [tiab] or gout [mh] or gout [tiab] |
| 3 | Randomized controlled trial [pt] or clinical trial [pt] or controlled clinical trial [pt] |
| 4 | Double-blind method [mh] or single-blind method [mh] or random allocation [mh] |
| 5 | Randomized controlled trials as topic [mh] or clinical trials as topic [mh] or controlled clinical trials as topic [mh] |
| 6 | Compare [tiab] or comparison [tiab] or compared [tiab] or comparing [tiab] |
| 7 | Parallel group [tiab] or parallel groups [tiab] |
| 8 | Crossover [tiab] or cross-over [tiab] or cross over [tiab] |
| 9 | Random [tiab] or randomized [tiab] or randomly [tiab] or randomization [tiab] |
| 10 | Randomized control [tiab] or clinical trial [tiab] or controlled trial [tiab] or control group [tiab] or placebo [tiab] |
| 11 | Trial [ti] |
| 12 | Single blind [tiab] or single blinded [tiab] or single blindly [tiab] or singly blind [tiab] or singly blinded [tiab] or double blind [tiab] or double blinded [tiab] or double blindly [tiab] or doubly blind [tiab] or doubly blinded [tiab] |
| 13 | Open label [tiab] or open-label [tiab] |
| 14 | Assign [tiab] or assigned [tiab] or assignment [tiab] or allocate [tiab] or allocated [tiab] or allocation [tiab] or match [tiab] or matched [tiab] or alternate [tiab] or alternative [tiab] |
| 15 | Cross-over studies [mh] |
| 16 | Placebo [tiab] or control group [tiab] |
| 17 | 3 or 4 or 5 or 6 or 7 or 8 or 9 or 10 or 11 or 12 or 13 or 14 or 15 or 16 |
| 18 | 1 and 2 and 16 and 17 |
| 19 | Animals [mh] not (animals [mh] and humans [mh]) |
| 20 | Systematic review [pt] |
| 21 | 19 or 20 |
| 22 | 18 not 21 |

## Supplementary Table 3. Search strategy for Embase. Medical subject heading (/), title/abstract (.ti,ab), title (ti).

| **Embase** | |
| --- | --- |
| 1 | Losartan/ or Losartan potassium/ |
| 2 | Losartan.ti,ab. |
| 3 | 1 or 2 |
| 4 | uric acid/ or urate/ or gout/ |
| 5 | (uric acid or urate or gout).ti,ab. |
| 6 | 4 or 5 |
| 7 | randomized controlled trial/ or controlled clinical trial/ or clinical trial/ |
| 8 | double blind procedure/ |
| 9 | single blind procedure/ |
| 10 | randomization/ |
| 11 | random allocation/ |
| 12 | controlled study/ |
| 13 | (placebo or control group).ti,ab. |
| 14 | (compare or comparison or comparing or compared).ti,ab. |
| 15 | (parallel group or parallel groups).ti,ab. |
| 16 | (crossover or cross-over or cross over).ti,ab. |
| 17 | (random or randomized or randomly or randomization).ti,ab. |
| 18 | ((allocate or allocated or allocation or assign or assigned or assignment or match or matched) adj5 (participant or participants or person or people or alternate or alternative or subject or subjects or group or groups or patient or patients or volunteer or volunteers)).ti,ab. |
| 19 | (randomized control or control group or clinical trial or controlled trial).ti,ab. |
| 20 | trial.ti. |
| 21 | ((single or singly or double or doubly) adj (blind or blindly or blinded)).ti,ab |
| 22 | (assigned or allocated).ti,ab. |
| 23 | (controlled adj7 (study or design or trial)).ti,ab. |
| 24 | (open label or open-label).ti,ab. |
| 25 | 7 or 8 or 9 or 10 or 11 or 12 or 13 or 14 or 15 or 16 or 17 or 18 or 19 or 20 or 21 or 22 or 23 or 24 |
| 26 | (systematic review not (trial or study)).ti |
| 27 | Animal experiment/ not (human experiment/ or human/) |
| 28 | 26 or 27 |
| 29 | 3 and 6 and 13 and 25 |
| 30 | 29 not 28 |

## Supplementary Table 4. Search strategy for Cochrane. Medical subject heading (MeSH); Title, abstract, keyword (ti,ab,kw).

| **Cochrane** | |
| --- | --- |
| 1 | MeSH descriptor: [Losartan] explode all trees |
| 2 | ("losartan"):ti,ab,kw |
| 3 | 1 or 2 |
| 4 | MeSH descriptor: [Uric Acid] explode all trees |
| 5 | (uric acid):ti,ab,kw |
| 6 | (urate):ti,ab,kw |
| 7 | MeSH descriptor: [Gout] explode all trees |
| 8 | (gout):ti,ab,kw |
| 9 | 4 or 5 or 6 or 7 or 8 |
| 10 | ("placebo"):ti,ab,kw |
| 11 | (control group):ti,ab,kw |
| 12 | 10 or 11 |
| 13 | 3 and 9 and 12 |

Supplementary Table 5. Summary table of trials included in the meta-analysis. Mean (standard deviation) shown. N – sample size who completed the study, NR – not reported. *Estimated values calculated using data from losartan and placebo group, to reflect the total sample size who completed the study. †Baseline demographic values reported for 193 and 188 participants initially recruited in losartan and placebo group, respectively. ‡Baseline demographic values reported for 157 and 157 participants in losartan and placebo group, respectively. §Baseline demographic values reported for 34 and 32 participants initially recruited in losartan and placebo group, respectively. ¶Randomised cross-over trial.

|  |  |  |  |  | **Total sample** | | | **Losartan** | | | **Placebo** | | |
| --- | --- | --- | --- | --- | --- | --- | --- | --- | --- | --- | --- | --- | --- |
| **Study** | **Ethnicity** | **Dose of losartan** | **Losartan Indication** | **Treatment duration**  **(weeks)** | **N** | **Age** | **Male (%)** | **Age** | **Male (%)** | **Urate baseline (mg/dL)** | **Age** | **Male (%)** | **Urate baseline (mg/dL)** |
| Flack, 2001 | African American | 50-150 mg | Mild to moderate hypertension | 12 | 339 | 50.5 (10.3)* | 43.1* | 50.4 (10.5)^†^ | 45.1^†^ | 4.9 | 50.6 (10.2)^†^ | 41.0^†^ | NR |
| Saruta, 2007 | Japanese | 50 mg | Essential hypertension | 8 | 319 | 55.2 (10.2)* | 58.3* | 55.5 (9.9)^‡^ | 61.8^‡^ | 5.8 | 54.8 (10.5)^‡^ | 54.8^‡^ | 5.8 |
| Nouri-Majalan, 2009 | Persian | 50 mg | Post renal transplant | 52 | 50 | 40.0 (13.0) | 60.0 | NR | NR | 6.2 | NR | NR | 6.8 |
| Zhu, 2009 | Chinese | 50 mg | Post renal transplant | 26 | 52 | 40.4 (11.5)* | 65.0* | 40.6 (11.6)^§^ | 67.6^§^ | 7.8 | 40.3 (11.6)^§^ | 62.5^§^ | 7.3 |
| Smink, 2012 | Multi-ethnic | 50-100 mg | Diabetic nephropathy | 26 | 1342 | 60.1 (7.4)* | 62.8 | 60.0 (7.3) | 62.2 | 6.7 | 60.2 (7.6) | 63.4 | 6.7 |
| Castillo-Ojo, 2023^¶^ | American | 50 mg | Hypertension | 4 | 110 | 52.3 (9.3) | 41.8 | 52.3 (9.3) | 41.8 | 5.2 | 52.3 (9.3) | 41.8 | 5.2 |

## Supplementary Table 6. Characteristics of the initial control group and the timing-eligible control subset. Data are counts and percentages or means and standard deviations. BMI – body mass index, eGFRcrea – estimated glomerular filtration rate based on serum creatinine.

| Variable | Initial group | Timing-eligible group | Standardised  mean  difference |
| --- | --- | --- | --- |
| n | 25,550 | 6,599 | NA |
| Age (years) | 57.44 (7.48) | 58.38 (7.26) | 0.127 |
| BMI (kg/m^2^) | 27.55 (4.79) | 28.53 (5.13) | 0.197 |
| Sex (male) | 13,133 (51.4%) | 3,675 (55.7%) | 0.086 |
| Alcohol intake (daily or almost daily) | 5,758 (22.5%) | 1,589 (24.1%) | 0.037 |
| Alcohol intake (three or four times a week) | 6,505 (25.5%) | 1,620 (24.5%) | 0.021 |
| Alcohol intake (once or twice a week) | 6,480 (25.4%) | 1,671 (25.3%) | 0.001 |
| Alcohol intake (one to three times a month) | 2,616 (10.2%) | 646 (9.8%) | 0.015 |
| Alcohol intake (special occasions only) | 2,503 (9.8%) | 622 (9.4%) | 0.013 |
| Alcohol intake (never) | 1,688 (6.6%) | 451 (6.8%) | 0.009 |
| Townsend deprivation index | -1.82 (2.83) | -1.84 (2.75) | 0.008 |
| Systolic blood pressure (mmHg) | 141.75 (19.73) | 144.44 (19.78) | 0.136 |
| eGFRcrea (ml/min/1.73 m^2^) | 88.73 (11.95) | 87.07 (13.06) | 0.133 |
| Type 2 diabetes (cases) | 1,240 (4.9%) | 437 (6.6%) | 0.076 |
| Gout (cases) | 2,970 (11.6%) | 1,700 (25.8%) | 0.369 |
| Diuretic use | 2,200 (8.6%) | 937 (14.2%) | 0.176 |
| Urate lowering therapy (allopurinol) | 984 (3.9%) | 691 (10.5%) | 0.259 |
| Urate lowering therapy (febuxostat) | 26 (0.1%) | 19 (0.3%) | 0.042 |
| Urate lowering therapy (sulfinpyrazone) | 4 (0.0%) | 3 (0.0%) | 0.017 |
| Urate - baseline measurement (mg/dL) | 5.36 (1.47) | 5.74 (1.72) | 0.236 |

## Supplementary Table 7. Characteristics of the initial treatment group and the timing-eligible treated subset. Data are counts and percentages or means and standard deviations. BMI – body mass index, eGFRcrea – estimated glomerular filtration rate based on serum creatinine.

| Variable | Initial group | Timing-eligible group | Standardised  mean  difference |
| --- | --- | --- | --- |
| n | 741 | 35 | NA |
| Age (years) | 60.3 (6.6) | 62.2 (5.5) | 0.291 |
| BMI (kg/m^2^) | 29.5 (4.9) | 29.5 (4.0) | 0.001 |
| Sex (male) | 397 (53.6%) | 25 (71.4%) | 0.369 |
| Alcohol intake (daily or almost daily) | 158 (21.3%) | 5 (14.3%) | 0.184 |
| Alcohol intake (three or four times a week) | 162 (21.9%) | 15 (42.9%) | 0.449 |
| Alcohol intake (once or twice a week) | 175 (23.6%) | 7 (20.0%) | 0.088 |
| Alcohol intake (one to three times a month) | 84 (11.3%) | 2 (5.7%) | 0.201 |
| Alcohol intake (special occasions only) | 96 (13.0%) | 4 (11.4%) | 0.047 |
| Alcohol intake (never) | 66 (8.9%) | 2 (5.7%) | 0.123 |
| Townsend deprivation  index | -1.5 (3.0) | -2.0 (2.7) | 0.148 |
| Systolic blood pressure (mmHg) | 155.9 (18.8) | 160.0 (18.9) | 0.219 |
| eGFRcrea (ml/min/1.73 m^2^) | 84.5 (14.4) | 80.9 (17.2) | 0.246 |
| Type 2 diabetes (cases) | 80 (10.8%) | 4 (11.4%) | 0.020 |
| Gout (cases) | 154 (20.8%) | 15 (42.9%) | 0.474 |
| Diuretic use | 442 (59.6%) | 17 (48.6%) | 0.222 |
| Urate lowering therapy (allopurinol) | 93 (12.6%) | 11 (31.4%) | 0.456 |
| Urate lowering therapy (febuxostat) | 2 (0.3%) | 1 (2.9%) | 0.209 |
| Urate lowering therapy (sulfinpyrazone) | 646 (87.2%) | 23 (65.7%) | 0.506 |
| Urate - baseline measurement (mg/dL) | 5.78 (1.58) | 6.38 (1.79) | 0.381 |

## Supplementary Table 8. Effects of losartan on serum urate from the leave-one-out analysis of the UK Biobank target trial emulation. Effect – the mean change in serum urate (mg/dL) in losartan-treated individuals relative to controls, 95% CIL – 95% confidence interval lower boundary, 95% CIU – 95% confidence interval upper boundary, P-value – level of statistical significance.

| Iteration | Effect | 95%CIL | 95%CIU | P-value |
| --- | --- | --- | --- | --- |
| 1 | -0.4 | -0.708 | -0.092 | 0.011 |
| 2 | -0.36 | -0.696 | -0.024 | 0.036 |
| 3 | -0.38 | -0.692 | -0.068 | 0.017 |
| 4 | -0.33 | -0.661 | 0.001 | 0.051 |
| 5 | -0.3 | -0.613 | 0.013 | 0.06 |
| 6 | -0.34 | -0.677 | -0.003 | 0.048 |
| 7 | -0.34 | -0.666 | -0.014 | 0.041 |
| 8 | -0.34 | -0.674 | -0.006 | 0.046 |
| 9 | -0.41 | -0.722 | -0.098 | 0.01 |
| 10 | -0.33 | -0.654 | -0.006 | 0.046 |
| 11 | -0.3 | -0.621 | 0.021 | 0.067 |
| 12 | -0.38 | -0.708 | -0.052 | 0.023 |
| 13 | -0.32 | -0.662 | 0.022 | 0.067 |
| 14 | -0.38 | -0.703 | -0.057 | 0.021 |
| 15 | -0.33 | -0.654 | -0.006 | 0.046 |
| 16 | -0.35 | -0.681 | -0.019 | 0.038 |
| 17 | -0.3 | -0.636 | 0.036 | 0.08 |
| 18 | -0.34 | -0.663 | -0.017 | 0.039 |
| 19 | -0.3 | -0.617 | 0.017 | 0.064 |
| 20 | -0.28 | -0.588 | 0.028 | 0.075 |
| 21 | -0.39 | -0.724 | -0.056 | 0.022 |
| 22 | -0.32 | -0.636 | -0.004 | 0.047 |
| 23 | -0.35 | -0.686 | -0.014 | 0.041 |

## Supplementary Table 9. Effects of losartan on serum urate with additional adjustment for renal function and diuretic use in the UK Biobank target trial emulation – sensitivity analyses. eGFRcrea – estimated glomerular filtration rate based on serum creatinine, Effect – the mean change in serum urate (mg/dL) in losartan-treated individuals relative to controls, 95% CIL – 95% confidence interval lower boundary, 95% CIU – 95% confidence interval upper boundary, P-value – level of statistical significance.

| Model | Covariates added | Effect | 95% CIL | 95% CIU | P-value |
| --- | --- | --- | --- | --- | --- |
| Primary | Baseline | -0.35 | -0.66 | -0.03 | 0.03 |
| Sensitivity 1 | eGFRcrea | -0.35 | -0.69 | -0.02 | 0.041 |
| Sensitivity 2 | Diuretic use | -0.34 | -0.68 | -0.001 | 0.049 |
| Sensitivity 3 | eGFRcrea and Diuretic use | -0.34 | -0.69 | 0.01 | 0.059 |
